# Tuberculosis Control and Role of Molecular Epidemiology Studies in Iran: A Systematic Review

**Published:** 2017

**Authors:** Mahya Pourostadi, Jalil Rashedi, Behroz Mahdavi Poor, Hossein Samadi Kafil, Abdolhassan Kazemi, Mohammad Asgharzadeh

**Affiliations:** 1 Hematology and Oncology Research Center, Tabriz University of Medical Sciences, Tabriz, Iran.; 2 Tuberculosis and Lung Diseases Research Center, and Department of Laboratory Science, Faculty of Paramedicine, Tabriz University of Medical Sciences, Tabriz, Iran.; 3 Department of Laboratory Science, Faculty of Paramedicine, Tabriz University of Medical Sciences, Tabriz, Iran.; 4 Drug Applied Research Center, Tabriz University of Medical Sciences, Tabriz, Iran.; 5 Infectious and Tropical Diseases Research Center, Tabriz University of Medical Sciences, Tabriz, Iran.; 6 Biotechnology Research Center, and Department of Laboratory Science, Faculty of Paramedicine, Tabriz University of Medical Sciences, Tabriz, Iran.

**Keywords:** Molecular epidemiology, Restriction Fragment Length Polymorphisms (RFLP), Tuberculosis, Transmission, Control, Iran

## Abstract

**Background:**

Today because of immigration, HIV pandemic and spread of drug resistant tuberculosis, mortality caused by tuberculosis (TB) has increased. To control the disease it is essential to identify the sources of the infection and patterns of transmission. This becomes possible through using molecular epidemiology methods.

**Materials and Methods:**

This research reviewed studies based on IS6110-restriction fragment length polymorphism (IS6110-RFLP) as a primary method to recognize the role of molecular epidemiology researches in managing TB in Iran. Within 1995–2015 all human population based studies which had use IS6110-RFLP as the primary method systematically reviewed.

**Results:**

At the end, 11 articles were selected. The mean clustering rate obtained was 28.1%. The rate of recent transmission was variable from 2% to 49% and the mean value was determined 17%.

**Conclusion:**

The studies revealed that in Iran both reactivation and recent transmission were significant in developing new cases of TB. Yet, reactivation plays greater role. If the matter is supervised insufficiently and carelessly, because of increasing rate of drug resistant TB, immigration of HIV infected individuals, TB especially drug resistant TB will be problematic in the near future.

## INTRODUCTION

Despite access to anti-tuberculosis (TB) drugs and global attempts to manage the disease, people continue to be infected. Approximately one- third of the world’s population is infected with *Mycobacterium tuberculosis*. Yet in only 10% of cases are their clinical manifestations of the infection (1). Based on the World Health Organization (WHO) 2014 global TB report, the incidence of TB was 9.6 million people and there were 1.5 million deaths from TB. Of the people infected with TB, 400,000 were HIV positive (2). At present, TB control programs encounter three major obstacles: the HIV epidemic (3); the spread of drug resistance, particularly extensively drug-resistant tuberculosis (XDR-TB) (4–6); and increasing immigration (7). Methods capable of indentifying TB transmission patterns, especially multidrug-resistant tuberculosis (MDR-TB), will aid in control of the disease.

Planning TB control programs requires identifying the sources of the infection (8), screening to prevent the spread of the disease (9), and treatment of individuals infected with TB. Identification of transmission patterns is possible using molecular epidemiology approaches, including molecular typing methods, in conjunction with classical epidemiologic methods. This approach enhances our knowledge of transmission routes and the spread of the infection (10). Additionally, we can distinguish recently transmitted TB from reactivated TB (11).

Molecular epidemiology studies using molecular typing methods can evaluate transmission patterns and risk factors, identify transmitted cases of MDR-TB, and determine transmission patterns within human populations (12). Results of these studies will make it possible to prevent the disease. Over the last two decades molecular epidemiology studies to control and prevent TB have been conducted in Iran, and the number of TB cases has significantly decreased. In molecular epidemiology the standard identification method is the IS6110-based restriction fragment length polymorphism (RFLP). This method is based on the numbers and genomic sites of the IS6110 fragment (13). As the copy numbers of this sequence differ from one strain to another (0–25 copy number) (14), it is a useful tool in TB epidemiology research and a method to track TB transmission (15,16).

The present study is a systematic review of previous research using the IS6110-RFLP method in Iran. The goal is to recognize the role of molecular epidemiology in the control of TB, which have been neglected, and to reduce the rate of the disease in Iran.

## MATERIALS AND METHODS

### Study selection

This survey consisted of molecular epidemiology studies of different Iranian populations. Inclusion criteria were: IS6110-RFLP as the primary fingerprinting method; more than 57 cases; a study period of six months or more; and identification of the number of isolates within each cluster.

Research that used other primary methods; specific populations, such as patients with MDR-TB or HIV; or cases infected with specific strains, were excluded.

### Literature search

The electronic databases PubMed, SCOPUS, Google Scholar and Scientific Information Database were searched from 1995 until the end of 2015. The keywords of tuberculosis, molecular epidemiology, IS6110-RFLP, and Iran were selected to find articles in both Farsi and English.

### Data extraction

Data collected included: the duration of the study; the time; region; sample size; genotyping method; clustering proportions; cluster size; the secondary typing method for strains with few IS6110 bands (if done); and risk factors. To calculate the ratio of TB due to recent transmission, to the total number of infected patients, the formula below was used (17). The formula assumes that each cluster is of an infected source in which the disease has become active and the others members of the cluster have recently acquired the disease from that source.

$$\begin{array}{c} \mathrm{Minimum}\;\mathrm{estimated}\;\mathrm{rate}\;\mathrm{of}\;\mathrm{recent}\;\mathrm{transmission}\;=\\ \frac{\mathrm{Number}\;\mathrm{of}\;\mathrm{clustered}\;\mathrm{patients}-\mathrm{Number}\;\mathrm{of}\;\mathrm{clusters}}{\mathrm{Total}\;\mathrm{number}\;\mathrm{of}\;\mathrm{patients}} \end{array}$$

Isolates which had a unique pattern were categorized as non-clustered and those with the same IS6110-RFLP pattern as clustered.

## RESULTS

**Figure 1:**
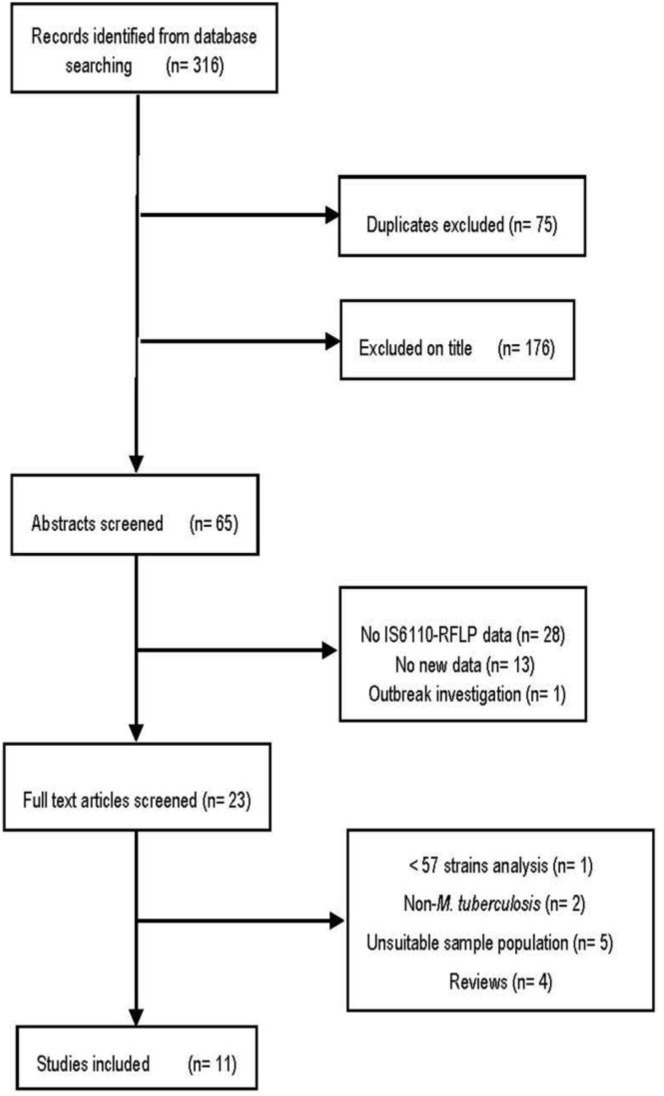
Flow diagram of literature review process.

The published articles related to the molecular epidemiology of TB were reviewed. Articles that did not conduct DNA fingerprinting; did not use IS6110-RFLP as the primary method; or did not mention cluster size were excluded. Finally, the data from 11 articles was evaluated (Figure 1)(18–28).

**Figure 2:**
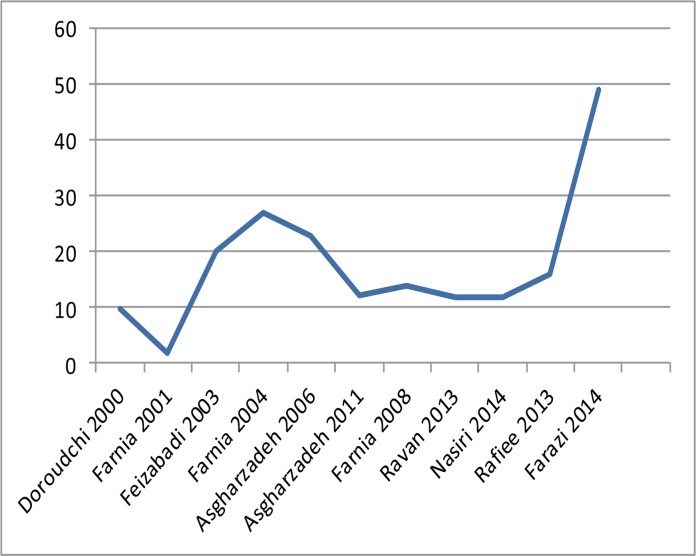
Minimum estimated rate of recent tuberculosis transmission in 11 studies

Extracted data is summarized in table 1. In eight studies secondary fingerprinting methods, including mycobacterial interspersed repetitive unit-exact tandem repeat (MIRU-ETR), spoligotyping, polymorphic guanine cytosine-rich repetitive sequence (PGRS-RFLP), and direct repeat (DR-RFLP), were used. Three studies lacked the secondary fingerprinting methods(19,22, 25). One study was deleted because the few copies of IS6110-RFLP affected the clustering (19). Overall, 0.5% of the isolates were excluded because a secondary fingerprinting method was not done, few copies of IS6110 were identified, there was laboratory contamination, or a non-*M. tuberculosis* infection was identified. The average rate of clustering was 28.1%. The maximum number of isolates within a cluster was eight, seen in East Azarbaijan province. These isolates were from Iranians (22). The maximum clustering rate of 78.9% was seen in Markazi province,(28) and the recent transmission rate was 49% [(75−28)/95] (Figure 2). The rate of acquiring an infection increased compared to a previous study in this area. The risk factors within the cluster were not evaluated in five studies (18,19,25–27). In three studies the risk factors were not significant,(20,23, 24) while in the other three studies some of the risk factors were significant (21,22, 28). Risk factors were: close contact and low age (two studies);(21,28) male sex and intravenous drug abuse (one study);(21) and a new case and degree of sputum smear (one case)(28). In a study in North West Iran, the isolates without resistance were significantly within the cluster (22).

**Table 1:** Selected characteristics of included studies

**No**	**First author**	**Study region**	**Study year**	**Study duration (months)**	**Study subjects**	**Isolates included in clusters**	**Unique patterns**	**Cluster No.**	**Secondary genotyping method**	**Cluster size**	Clustered % ^*^
1	Doroudchi, et al, 2000 (18)	Tehran and Fars	1995–96 and 97	12	62	12	50	6	Spoligotyping	2	19
2	Farnia, et al, 2001 (19)	East and West Azerbaijan, Khorasan, Kerman, Kermanshah and Fars	1998–99	6	62	2	60	1	-	2	3
3	Feizabadi, , et al, 2003 (20)	Tehran	1999–2000	18	120	33	86	9	PGRS-RFLP	2–6	28
4	Farnia, et al. 2004 (21)	Tehran	2001	12	129	56	73	21	Spoligotyping	2–7	43
5	Asgharzadeh, et al, 2006 (22)	East Azarbaijan	2002–2003	6	105	35	70	11	-	2–8	33
6	Asgharzadeh, et al, 2011 (23)	East and West Azerbaijan	2004–2005	12	154	32	122	13	MIRU-VNTR	2–6	21
7	Farnia, et al, 2008 (24)	Tehran	2006–2007	12	258	65	193	29	Spoligotyping	2–3	25
8	Ravan, et al, 2013 (25)	Tehran, Mashhad, Isfahan, Shiraz and Ahvaz	-	-	258	61	197	30	-	2–3	24
9	Nasiri, et al, 2014 (26)	Tehran	2008–2009	24	291	60	231	25	Spoligotyping	2–5	21
10	Rafiee, et al, 2013 (27)	Markazi	2010–2011	12	57	16	41	7	DR-RFLP	2–3	28
11	Farazi, et al, 2014 (28)	Markazi	2011–2012	18	95	75	20	28	PGRS-RFLP	2–6	78.9

**Note:**

* Number of subjects in TB cluster/ Number of all subjects. PGRS, polymorphic guanine cytosine – rich repetitive sequence; RFLP, restriction fragment length polymorphism; DR, direct repeat.

## DISCUSSION

Over the last 23 years molecular epidemiology studies have answered questions which had remained unanswered for many years. The TB burden can be attributed to the recent transmission or reactivation. Determining recent transmission rates and identifying highly transmissible strains can decrease the transmission cycle. The recent studies show that accurately estimating the TB transmission rate by traditionally methods is not possible (29). Yet, DNA fingerprinting can determine the TB transmission cycle and the associated risk factors. Eleven reviewed population-based studies in Iran that used the IS6110-RFLP method indicated the rate of recent transmission was 2–49%, with an average of 17% ($\frac{447-180}{1591}=0.17$) (Figure 2). This suggests that in Iran the majority of TB cases are due to reactivation rather than recent transmission. Although urbanization has increased in Iran, and immigrants, particularly from Afghanistan, inhabit in the margins of the large cities, the majority of strains have a unique pattern. Over 83% of cases occur due to reactivation. The rate of recent transmission differs in different places and at different times. In Doroudchi et al (18) study, the rate of recent transmission was low (9.7%). Studies with a greater number of isolates provide more reliable results (21,23). Subsequent studies showed a decline in transmission (24,25, 27), which was in agreement with the reduction of TB prevalence in the country.

In the latest study in Iran using the IS6110-RFLP method, the transmission rate was 49% (28), which is high compared with earlier studies. As in our study, the smear-positive samples were included and these cases had a role in transmitting the infection. The majority of these cases were new, not reinfection. Therefore, the percentage of the recent transmission was higher. A low variety of strains of *M. tuberculosis* in the study region and high degree of close contact among people can increase the rate of recent transmission. It must be mentioned that the uniform fingerprinting patterns are not always indicative of definitive epidemiologic correlation. In some cases, the isolates from two individuals living far apart and without any contact have a similar pattern (30). This suggests that some strains may be more prevalent, which may cause confusion in epidemiologic correlations.

The average rate of clustering was 28.1%. The lowest rate was 3%(19) and the highest rate was 78.9% (28). The factors affecting the clustering rate are local TB incidence; new cases; the presence of strains with low IS6110 bands; sampling fraction (31); age of the subjects; and study duration. With an increase in the duration of a study, the rate of clustering in a population increases. In three to four years clustering reaches a plateau (32,33). In studies by Farnia et al.(19), and Storla et al. (34), the duration of the study was short and the clustering rate was low. Secondary typing methods were not used in the Farnia’s study,(19) and 38 isolates with low bands were excluded (as the IS6110 accuracy in the isolates with low bands is low). In the 62 remaining isolates, because of high number IS6110 bands, the rate of clustering was low.

In older individuals, TB is associated with the reactivation, and increasing the age of the study subjects decreases the clustering rate. In a study from Japan (35), the average age was 69.4 years which decreased the clustering rate. In contrast, the rate increases with subjects of younger age (36,37). For example, in a study from Tehran,(21) subjects were young, and the rate of clustering was higher (43%) (Table 1).

In our reviewed studies, the most common cluster size was two. The most crowded cluster consisted of eight isolates from patients living in East Azarbaijan province of Iran (22). Similarly, in a study from Belgrade, the most common cluster size was two and the largest cluster size was six (38). In a study from San Francisco (39), the largest cluster had 30 members. In Zimbabwe (40), a unique genotype was isolated from 116 patients (approximately half of the subjects).

Among the factors in increasing cluster size are emerging strains unique to a region (41). As unique strains are not common in Iran, the size of clusters was small. Thus, we can say the occurrence of TB in Iran is microepidemic. Since the maximum duration of the study in Iran was 24 months, most of the infected individuals in the study regions were not detected. Hence, the size of clusters was small. In Iran the population of intravenous drug abuser, alcohol consumers, homeless individuals, and HIV positive patients is low, and the size of clusters is small.

The effect of immigrants on the epidemiology of TB in developed countries has been evaluated. In Western European countries, among immigrants and foreign born groups, TB is often the result of recent transmission (42–43). Although the risk of TB transmission from immigrants to native people is low (44), the presence of TB infected immigrants increases the disease prevalence. Immigrants may be highly stressed, exposed to poverty, malnutrition, and confined in crowded places with poor conditions. These conditions increase the possibility of reactivation of the dormant bacteria. The possibility that immigrants acquire TB infection with a new strain of the bacteria is also higher. The prevalence of TB among Afghan immigrants in Iran is high (24,26), and the rate of MDR-TB is higher in Afghan immigrants compared to native Iranians (18,24, 25). This results in problems in the management of TB. Therefore, monitoring and treatment of immigrants, particularly those with MDR-TB, should be considered, as has been done in the Switzerland (42) and Netherlands (45).

Of the 11 reviewed articles, there were non-Iranian people in nine studies. Non-Iranians were not evaluated in two studies and a significant number of non-Iranians were included in seven studies. Afghans and other immigrants who come to Iran for TB treatment do not have an effective role in recent transmission. Considering that MDR-TB (25,26) and the Beijing strain (18,24) are more prevalent among immigrants than Iranians, and that the Beijing strain is highly transmissible, (41,46) monitoring and treatment of Afghan immigrants should be undertaken.

Genotyping methods can also determine nosocomial transmission, laboratory cross-contamination (47), and recurrent TB caused by reinfection. There is high reinfection rate in regions with a high TB burden (48). In areas where TB prevalence is low, reinfection rarely occurs. However, among TB susceptible populations, including patients with HIV/AIDS, recurrence of TB is mainly caused by reinfection (11,49).

The discrimination power of IS6110-RFLP is the most accurate, followed by MIRU-VNTR, spoligotyping, random-amplified polymorphic DNA- polymerase chain reaction (RAPD-PCR), and PFGE (50). The disadvantages of IS6110-RFLP include expense; time (several weeks) required for the bacterial growth; requirement for large amounts of pure DNA; low discrimination power in strains with fewer than six IS6110 bands (51); and comparison of results between laboratories is difficult (52). Therefore, in the case of few IS6110 bands (<6), a secondary genotyping method is recommended. The use of IS6110-RFLP in an individual with recent and recurrent infection may lead to misdiagnosis. It should be noted that in multiple infections caused by several strains, the intensity of the band color varies (53).

Ten percent of infected people develop clinical disease and there are risk factors that increase susceptibility *M. tuberculosis* infection. Risk factors include: close contact with TB infected individuals; living in cities; the number of people in the home; lack of social services and medical facilities (11); smoking; imprisonment; immigration (7, 12); young age (54); malnutrition; drug and alcohol abuse; homelessness (55,56); and HIV infection (3,17, 56). Risk factors differ among different population in Iran. Identification of risk factors will aid in the development of preventive strategies to target the populations at greatest risk. In Iran crowded places like coffee shops, mosques, buses, and subway trains may play role in transmitting the disease. In the capital city of Iran, a metropolitan city with many immigrants, being male and young are risk factors (21). This is due to more frequent contact and communication with other people and the relatively high prevalence of HIV among the youth population.

Four studies considered HIV infection as a risk factor. Overall 22 people were HIV positive, but it was not significant (p>0.05). However, in some cases there was no difference between sex and transmission of TB(23). This may be due to the high rate of unemployment and relatively high levels of poverty among elderly females in the region. In all reviewed studies the majority of people with TB, inside or outside of the cluster, lived in poor conditions.

In East Azarbaijan province, like Los Angeles, USA, the transmission rate of drug resistant TB was low (57). While in Estonia (4), the Archangel Oblast Region of Russia (58), and New York City (36), the drug resistant TB was more frequently transmitted. This occurred in New York due to poor management of TB control programs, the HIV epidemic, and the economical and social problems of the city(56). In Russia and Estonia the prevalence of the Beijing strain,(4) a strain with higher virulence, played a role (59). and the majority of drug resistant strains were this genotype (60). Four studies used the spoligotyping method (Table 1). Seventeen cases of the Beijing strain were reported, and eight were from Afghan immigrants. In East Azarbaijan the Beijing strain is uncommon, the prevalence of HIV is low, (61) and drug resistant TB transmission is low.

The goal of the TB control program is to eliminate the disease by breaking the transmission cycle. To reach this goal, rapid diagnosis and effective treatment is required. From the 1960’s, with the introduction of effective anti-TB drugs, the spread of disease in developed countries has decreased. However, from the late 80’s, due to insufficient supervision of TB control programs, and the HIV epidemic, the burden of the disease increased. In the Western Europe and North America TB was controlled within 20 years of interruption of the transmission cycle. With all the progresses in the field of prevention, diagnosis, and treatment of TB, unfortunately, based on the 2015 WHO report in some countries such as South Africa, Mozambique, Indonesia and Cambodia the rate of disease was more than 350 in one hundred thousand (2). In recent years, TB control programs have been successful in Iran. The prevalence rate of 35 per 100,000 in 1995, dropped to 20 in 100,000 in 2010. This progress was due to activities and studies in the transmission and the prevention of the infection. Unfortunately, in 2014, the decreasing prevalence did not continue, and the prevalence rate increased to 22 in 100,000(2). This may be due to the increased prevalence of HIV infection (62), improvement in reporting the cases to health centers, introduction of specific strains by immigrants, and travelers to Iran from Pakistan, Afghanistan (21) and Azerbaijan (16) for therapeutic purposes. The frequency of the Beijing strain in the Iranian population is low (nine cases). The existence of this strain in Iranians is likely due to the prevalence of this strain in Afghan immigrants (63,64). One of the most important characteristics of this genotype is its rapid distribution in the populations (41, 60, 65) and immunization with bacilli Calmette-Guerin (BCG) does not prevent disease caused by this genotype. Routine anti-TB medications are not effective in eliminating the Beijing genotype (59). Therefore, it is essential to determine the dynamics of the transmission to control this strain. The prevalence of TB in HIV-positive patients is higher than that in HIV negative people. The number of HIV-positive patients in Iran is increasing. Therefore, HIV-positive patients in Iran should be provided with education about the hazards TB and encouraged to seek medical care and to comply with treatment. Immigrants with dormant infection play a role in the transmission of disease (66), and Afghan immigrants with dormant infection should be identified and treated to prevent further dissemination of TB. Because of the role of crowded places, such as buses and subway trains, in transmission of the infection, daily disinfection of vehicles is recommended.

There are limitations in reviewed studies. The same methodology was not used in all studies. Duration of the studies was 24 months or less, whereas, 36–48 months would achieve optimum results in clustering and risk factors evaluation. In some studies risk factors were not evaluated, and when identified risk factors were often different. These methodological shortcomings create bias in the results. A uniform questionnaire in the countrywide health care system was not used. Therefore, the preparation of a uniform and comprehensive questionnaire is necessary to enhance the quality of TB research control programs. Another limitation was dividing the subjects into “clustered” and “non-clustered” groups. It is possible that some people located inside the cluster have reactivated TB, and that people inside the cluster do not have any contact with infected people.

To accurately determine the recent TB transmission rate, it is recommended that specimens be collected during a three to four year period and the strains of TB identified. It also recommended that genotyping be used to identify the specific strains. With the identification and typing of special drug-resistance strains, the development of XDRTB and totally drug- resistant tuberculosis (TDR-TB) can be prevented. This would decrease the burden on the public health system. Identification of multiple infections will allow the correct estimation of reactivated TB and recent transmission possible.

## CONCLUSION

Molecular epidemic studies in Iran will enhance our understanding of epidemiologic factors involved in dissemination of TB in the country. These studies also clarify the effective elements on clustering, and the information can be used to develop TB control programs. New cases of TB, either due to reactivation or recent transmission, are important in Iran with reactivation playing a significant role.
